# (*E*)-2-Meth­oxy-6-[(5-methyl­isoxazol-3-yl)imino­meth­yl]phenol

**DOI:** 10.1107/S1600536808001888

**Published:** 2008-01-23

**Authors:** Ren-Gao Zhao, Jie Lu, Ji-Kun Li

**Affiliations:** a Department of Materials Science and Chemical Engineering, Taishan University, 271021 Taian, Shandong, People’s Republic of China; b Department of Applied Science and Technology, Taishan University, 271021 Taian, Shandong, People’s Republic of China

## Abstract

In the title mol­ecule, C_12_H_12_N_2_O_3_, the benzene and isoxazole rings form a dihedral angle of 5.9 (6)°. The hydr­oxy group is involved in an intra­molecular O—H⋯N hydrogen bond [O⋯N = 2.616 (5) Å], resulting in approximate planarity of the mol­ecular skeleton. In the crystal structure, mol­ecules related by translation along the *c* axis are stacked into columns, the shortest inter­molecular C⋯C distance being 3.298 (6) Å.

## Related literature

For related crystal structures, see Li *et al.* (2007 ▶). For general background, see Garnovskii *et al.* (1993 ▶).
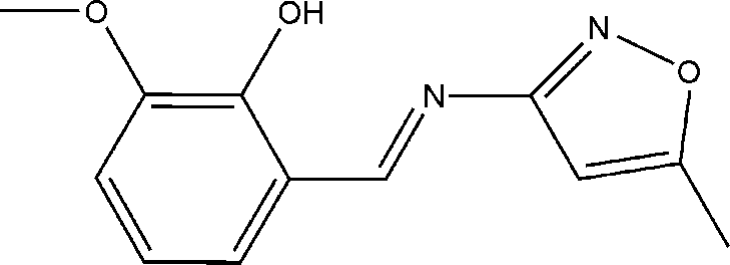


## Experimental

### 

#### Crystal data

C_12_H_12_N_2_O_3_
*M* *_r_* = 232.24
Orthorhombic, 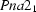

*a* = 22.254 (5) Å
*b* = 10.178 (5) Å
*c* = 4.836 (2) Å
*V* = 1095.4 (8) Å^3^
*Z* = 4
Mo *K*α radiation
μ = 0.10 mm^−1^
*T* = 273 (2) K
0.12 × 0.10 × 0.08 mm

#### Data collection

Bruker SMART CCD area-detector diffractometer
Absorption correction: multi-scan (*SADABS*; Sheldrick, 1996 ▶) *T*_min_ = 0.988, *T*_max_ = 0.992
3720 measured reflections
1079 independent reflections
651 reflections with *I* > 2σ(*I*)
*R*_int_ = 0.069

#### Refinement

*R*[*F* ^2^ > 2σ(*F* ^2^)] = 0.050
*wR*(*F* ^2^) = 0.122
*S* = 1.02
1079 reflections
156 parameters
H-atom parameters constrained
Δρ_max_ = 0.24 e Å^−3^
Δρ_min_ = −0.19 e Å^−3^

### 

Data collection: *SMART* (Siemens, 1996 ▶); cell refinement: *SMART*; data reduction: *SAINT* (Siemens, 1996 ▶); program(s) used to solve structure: *SHELXS97* (Sheldrick, 2008 ▶); program(s) used to refine structure: *SHELXL97* (Sheldrick, 2008 ▶); molecular graphics: *SHELXTL* (Sheldrick, 2008 ▶); software used to prepare material for publication: *SHELXTL*.

## Supplementary Material

Crystal structure: contains datablocks global, I. DOI: 10.1107/S1600536808001888/cv2374sup1.cif

Structure factors: contains datablocks I. DOI: 10.1107/S1600536808001888/cv2374Isup2.hkl

Additional supplementary materials:  crystallographic information; 3D view; checkCIF report

## Figures and Tables

**Table 1:** Hydrogen-bond geometry (Å, °)

*D*—H⋯*A*	*D*—H	H⋯*A*	*D*⋯*A*	*D*—H⋯*A*
O1—H1⋯N1	0.82	1.89	2.616 (5)	147

**Fig. 1.**
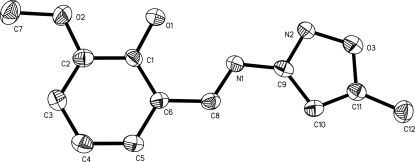
The molecular structure showing 30% probability displacement ellipsoids and the atom-numbering scheme.

## supplementary crystallographic information

### Comment

Recently, a number of Schiff-bases have been investigated in terms of their crystallography and coordination chemistry (Garnovskii *et al.*, 1993). In continuation of our studies of Schiff-bases, we report here the synthesis and crystal structure of the title compound, (I).

In (I) (Fig. 1), the geometric parameters are in good agreement with those found in 2,4-di-*tert*-butyl-6-(4-chlorophenyliminomethyl)phenol (Li *et al.*, 2007). The benzene and the isoxazole rings make a dihedral angle of 5.9 (6)°. The hydroxy group is involved in intramolecular O—H···N hydrogen bond (Table 2). In the crystal, the molecules related by translation along *c* axis are stacked into columns with the shortest intermolecular C···C distance of 3.298 (6) Å (Table 1), suggesting an existence of π···π interactions.

### Experimental

The title compound was synthesized by the reaction of 2-hydroxy-3-methoxybenzaldehyde (0.152 g, 1 mmol) and 5-methylisoxazol-3-amine (0.098 g, 1 mmol) in ethanol solution and stirred under reflux conditions (353 K) for 6 h. When cooled to room temperature the solution was filtered and after a week orange crystals suitable for X-ray diffraction study were obtained. Yield, 0.186 g, 80%. m.p. 365–367 K.

Analysis found: C 61.98, H 5.25, N 12.04%; C_12_H_12_N_2_O_3_ requires: C 62.02, H 5.21, N 12.06%.

### Refinement

The H atoms were geometrically positioned (C—H 0.93–0.96 Å, O—H = 0.82 Å) and refined in a riding-model approximation with *U*_iso_(H) = 1.2*U*_eq_(C-aromatic) and *U*_iso_(H) = 1.5*U*_eq_(*C*-methyl and O). Due to the absence of any significant anomalous scatterers in the molecule, the 758 Friedel pairs were merged before the final refinement.

### Crystal data

**Table:**

C_12_H_12_N_2_O_3_	*F*_000_ = 488
*M**_r_* = 232.24	*D*_x_ = 1.408 Mg m^−^^3^*D*_m_ = 1.408 Mg m^−^^3^*D*_m_ measured by not measured
Orthorhombic, *P**n**a*2_1_	Mo *K*α radiation λ = 0.71073 Å
Hall symbol: P 2c -2n	Cell parameters from 401 reflections
*a* = 22.254 (5) Å	θ = 2.7–18.4º
*b* = 10.178 (5) Å	µ = 0.10 mm^−^^1^
*c* = 4.836 (2) Å	*T* = 273 (2) K
*V* = 1095.4 (8) Å^3^	Block, orange
*Z* = 4	0.12 × 0.10 × 0.08 mm

### Data collection

**Table:**

Bruker SMART CCD area-detector diffractometer	1079 independent reflections
Radiation source: sealed tube	651 reflections with *I* > 2σ(*I*)
Monochromator: graphite	*R*_int_ = 0.069
*T* = 273(2) K	θ_max_ = 25.1º
φ and ω scans	θ_min_ = 1.8º
Absorption correction: multi-scan(SADABS; Sheldrick, 1996)	*h* = −26→12
*T*_min_ = 0.988, *T*_max_ = 0.992	*k* = −11→10
3720 measured reflections	*l* = −5→5

### Refinement

**Table:**

Refinement on *F*^2^	Hydrogen site location: inferred from neighbouring sites
Least-squares matrix: full	H-atom parameters constrained
*R*[*F*^2^ > 2σ(*F*^2^)] = 0.050	*w* = 1/[σ^2^(*F*_o_^2^) + (0.052*P*)^2^] where *P* = (*F*_o_^2^ + 2*F*_c_^2^)/3
*wR*(*F*^2^) = 0.122	(Δ/σ)_max_ < 0.001
*S* = 1.02	Δρ_max_ = 0.24 e Å^−^^3^
1079 reflections	Δρ_min_ = −0.19 e Å^−^^3^
156 parameters	Extinction correction: SHELXL97 (Sheldrick, 2008), Fc^*^=kFc[1+0.001xFc^2^λ^3^/sin(2θ)]^-1/4^
Primary atom site location: structure-invariant direct methods	Extinction coefficient: 0.009 (3)
Secondary atom site location: difference Fourier map	

### Special details

**Table:**

Geometry. All e.s.d.'s (except the e.s.d. in the dihedral angle between two l.s. planes) are estimated using the full covariance matrix. The cell e.s.d.'s are taken into account individually in the estimation of e.s.d.'s in distances, angles and torsion angles; correlations between e.s.d.'s in cell parameters are only used when they are defined by crystal symmetry. An approximate (isotropic) treatment of cell e.s.d.'s is used for estimating e.s.d.'s involving l.s. planes.
Refinement. Refinement of *F*^2^ against ALL reflections. The weighted *R*-factor *wR* and goodness of fit *S* are based on *F*^2^, conventional *R*-factors *R* are based on *F*, with *F* set to zero for negative *F*^2^. The threshold expression of *F*^2^ > σ(*F*^2^) is used only for calculating *R*-factors(gt) *etc*. and is not relevant to the choice of reflections for refinement. *R*-factors based on *F*^2^ are statistically about twice as large as those based on *F*, and *R*- factors based on ALL data will be even larger.

### Fractional atomic coordinates and isotropic or equivalent isotropic displacement parameters (Å^2^)

**Table:**

	*x*	*y*	*z*	*U*_iso_*/*U*_eq_	
O1	0.38927 (16)	0.1624 (3)	0.1475 (8)	0.0589 (12)	
H1	0.3605	0.1711	0.0433	0.088*	
O2	0.47481 (17)	0.1609 (3)	0.5178 (8)	0.0642 (12)	
O3	0.19927 (17)	0.2331 (3)	−0.6386 (9)	0.0630 (12)	
N1	0.30586 (18)	0.2894 (4)	−0.1253 (10)	0.0435 (11)	
N2	0.2451 (2)	0.1937 (4)	−0.4528 (11)	0.0600 (14)	
C1	0.3990 (2)	0.2760 (5)	0.2836 (10)	0.0439 (14)	
C2	0.4440 (2)	0.2775 (5)	0.4844 (12)	0.0493 (15)	
C3	0.4554 (2)	0.3904 (5)	0.6332 (11)	0.0507 (14)	
H3	0.4856	0.3913	0.7660	0.061*	
C4	0.4214 (2)	0.5035 (5)	0.5842 (12)	0.0521 (15)	
H4	0.4286	0.5792	0.6867	0.062*	
C5	0.3773 (2)	0.5031 (5)	0.3843 (12)	0.0488 (13)	
H5	0.3551	0.5790	0.3520	0.059*	
C6	0.3655 (2)	0.3901 (5)	0.2295 (10)	0.0408 (13)	
C7	0.5192 (3)	0.1552 (6)	0.7308 (13)	0.0709 (19)	
H7A	0.5505	0.2174	0.6920	0.106*	
H7B	0.5359	0.0683	0.7384	0.106*	
H7C	0.5009	0.1760	0.9054	0.106*	
C8	0.3197 (2)	0.3922 (5)	0.0215 (11)	0.0448 (13)	
H8	0.2990	0.4702	−0.0098	0.054*	
C9	0.2608 (2)	0.3021 (5)	−0.3245 (11)	0.0457 (14)	
C10	0.2266 (2)	0.4109 (5)	−0.4155 (12)	0.0512 (15)	
H10	0.2295	0.4971	−0.3536	0.061*	
C11	0.1892 (2)	0.3643 (5)	−0.6087 (11)	0.0464 (14)	
C12	0.1426 (2)	0.4195 (6)	−0.7923 (12)	0.0658 (18)	
H12A	0.1372	0.3628	−0.9489	0.099*	
H12B	0.1548	0.5050	−0.8544	0.099*	
H12C	0.1054	0.4266	−0.6928	0.099*	

### Atomic displacement parameters (Å^2^)

**Table:**

	*U*^11^	*U*^22^	*U*^33^	*U*^12^	*U*^13^	*U*^23^
O1	0.069 (3)	0.040 (2)	0.068 (3)	0.0036 (19)	−0.016 (2)	−0.009 (2)
O2	0.068 (3)	0.056 (2)	0.069 (3)	0.016 (2)	−0.019 (2)	−0.004 (2)
O3	0.070 (3)	0.049 (2)	0.070 (3)	−0.006 (2)	−0.013 (2)	−0.007 (2)
N1	0.045 (3)	0.036 (2)	0.049 (2)	−0.002 (2)	−0.002 (2)	−0.003 (2)
N2	0.068 (3)	0.041 (3)	0.071 (3)	−0.009 (2)	−0.027 (3)	0.002 (3)
C1	0.046 (3)	0.041 (3)	0.045 (3)	−0.009 (3)	−0.004 (3)	0.000 (3)
C2	0.049 (4)	0.044 (3)	0.056 (4)	0.003 (3)	0.007 (3)	−0.001 (3)
C3	0.048 (3)	0.055 (3)	0.049 (3)	−0.003 (3)	−0.002 (3)	−0.005 (3)
C4	0.060 (4)	0.044 (3)	0.052 (3)	−0.009 (3)	0.006 (3)	−0.002 (3)
C5	0.056 (3)	0.036 (3)	0.055 (3)	−0.001 (3)	0.000 (4)	−0.004 (3)
C6	0.041 (3)	0.034 (3)	0.047 (3)	0.002 (3)	0.003 (3)	0.001 (2)
C7	0.069 (4)	0.078 (4)	0.065 (4)	0.027 (3)	−0.011 (4)	−0.002 (4)
C8	0.042 (3)	0.038 (3)	0.055 (3)	0.004 (3)	0.004 (3)	0.000 (3)
C9	0.050 (4)	0.038 (3)	0.049 (3)	−0.007 (3)	0.008 (3)	−0.003 (3)
C10	0.056 (4)	0.039 (3)	0.059 (4)	0.007 (3)	−0.003 (3)	−0.007 (3)
C11	0.047 (4)	0.045 (3)	0.048 (3)	−0.001 (3)	0.007 (3)	−0.006 (3)
C12	0.064 (4)	0.073 (4)	0.061 (4)	0.008 (3)	−0.011 (4)	−0.005 (4)

### Geometric parameters (Å, °)

**Table:**

O1—C1	1.348 (5)	C4—H4	0.9300
O1—H1	0.8200	C5—C6	1.397 (7)
O2—C2	1.379 (6)	C5—H5	0.9300
O2—C7	1.428 (6)	C6—C8	1.433 (7)
O3—C11	1.362 (6)	C7—H7A	0.9600
O3—N2	1.417 (6)	C7—H7B	0.9600
N1—C8	1.301 (6)	C7—H7C	0.9600
N1—C9	1.397 (7)	C8—H8	0.9300
N2—C9	1.313 (5)	C9—C10	1.413 (6)
C1—C2	1.395 (7)	C10—C11	1.338 (7)
C1—C6	1.405 (7)	C10—H10	0.9300
C2—C3	1.379 (7)	C11—C12	1.477 (7)
C3—C4	1.397 (7)	C12—H12A	0.9600
C3—H3	0.9300	C12—H12B	0.9600
C4—C5	1.377 (7)	C12—H12C	0.9600
			
C4···C8^i^	3.298 (6)	C6···C9^i^	3.299 (6)
			
C1—O1—H1	109.5	O2—C7—H7B	109.5
C2—O2—C7	117.5 (4)	H7A—C7—H7B	109.5
C11—O3—N2	109.2 (4)	O2—C7—H7C	109.5
C8—N1—C9	118.2 (4)	H7A—C7—H7C	109.5
C9—N2—O3	104.7 (4)	H7B—C7—H7C	109.5
O1—C1—C2	117.7 (5)	N1—C8—C6	122.6 (5)
O1—C1—C6	122.2 (5)	N1—C8—H8	118.7
C2—C1—C6	120.1 (5)	C6—C8—H8	118.7
O2—C2—C3	124.4 (5)	N2—C9—N1	116.0 (5)
O2—C2—C1	115.4 (5)	N2—C9—C10	111.6 (5)
C3—C2—C1	120.2 (5)	N1—C9—C10	132.3 (5)
C2—C3—C4	119.9 (5)	C11—C10—C9	106.0 (5)
C2—C3—H3	120.0	C11—C10—H10	127.0
C4—C3—H3	120.0	C9—C10—H10	127.0
C5—C4—C3	120.1 (5)	C10—C11—O3	108.6 (5)
C5—C4—H4	119.9	C10—C11—C12	136.3 (5)
C3—C4—H4	119.9	O3—C11—C12	115.1 (5)
C4—C5—C6	120.8 (5)	C11—C12—H12A	109.5
C4—C5—H5	119.6	C11—C12—H12B	109.5
C6—C5—H5	119.6	H12A—C12—H12B	109.5
C5—C6—C1	118.8 (5)	C11—C12—H12C	109.5
C5—C6—C8	119.8 (5)	H12A—C12—H12C	109.5
C1—C6—C8	121.4 (5)	H12B—C12—H12C	109.5
O2—C7—H7A	109.5		
			
C11—O3—N2—C9	−0.8 (5)	O1—C1—C6—C8	−1.2 (7)
C7—O2—C2—C3	3.5 (8)	C2—C1—C6—C8	179.0 (4)
C7—O2—C2—C1	−176.8 (4)	C9—N1—C8—C6	−179.5 (4)
O1—C1—C2—O2	1.2 (7)	C5—C6—C8—N1	−178.6 (5)
C6—C1—C2—O2	−179.0 (5)	C1—C6—C8—N1	1.0 (7)
O1—C1—C2—C3	−179.0 (5)	O3—N2—C9—N1	179.6 (4)
C6—C1—C2—C3	0.8 (7)	O3—N2—C9—C10	0.8 (6)
O2—C2—C3—C4	−179.8 (5)	C8—N1—C9—N2	−175.0 (5)
C1—C2—C3—C4	0.5 (8)	C8—N1—C9—C10	3.5 (8)
C2—C3—C4—C5	−1.1 (8)	N2—C9—C10—C11	−0.5 (6)
C3—C4—C5—C6	0.5 (8)	N1—C9—C10—C11	−179.0 (5)
C4—C5—C6—C1	0.8 (7)	C9—C10—C11—O3	−0.1 (5)
C4—C5—C6—C8	−179.6 (4)	C9—C10—C11—C12	−178.7 (6)
O1—C1—C6—C5	178.4 (5)	N2—O3—C11—C10	0.6 (5)
C2—C1—C6—C5	−1.4 (7)	N2—O3—C11—C12	179.5 (4)

Symmetry codes: (i) *x*, *y*, *z*+1.

### Hydrogen-bond geometry (Å, °)

**Table:**

*D*—H···*A*	*D*—H	H···*A*	*D*···*A*	*D*—H···*A*
O1—H1···N1	0.82	1.89	2.616 (5)	147
